# Desertification of Iran in the early twenty-first century: assessment using climate and vegetation indices

**DOI:** 10.1038/s41598-021-99636-8

**Published:** 2021-10-15

**Authors:** Hadi Eskandari Dameneh, Hamid Gholami, Matt W. Telfer, Jesús Rodrigo Comino, Adrian L. Collins, John D. Jansen

**Affiliations:** 1 Department of Natural Resources Engineering, University of Hormozgan, Bandar-Abbas, Iran; 2 School of Geography, Earth and Environmental Sciences, Plymouth University, Plymouth, UK; 3 Departamento de Análisis Geográfico Regional y Geografía Física, Facultad de Filosofía y Letras, Campus Universitario de Cartuja, University of Granada, 18071 Granada, Spain; 4 Sustainable Agriculture Sciences Department, Rothamsted Research, North Wyke, Okehampton, EX20 2SB UK; 5 GFÚ Institute of Geophysics, Czech Academy of Sciences, Prague, Czech Republic

**Keywords:** Environmental sciences, Natural hazards

## Abstract

Remote sensing of specific climatic and biogeographical parameters is an effective means of evaluating the large-scale desertification status of drylands affected by negative human impacts. Here, we identify and analyze desertification trends in Iran for the period 2001–2015 via a combination of three indices for vegetation (NPP—net primary production, NDVI—normalized difference vegetation index, LAI—leaf area index) and two climate indices (LST—land surface temperature, P—precipitation). We combine these indices to identify and map areas of Iran that are susceptible to land degradation. We then apply a simple linear regression method, the Mann–Kendall non-parametric test, and the Theil–Sen estimator to identify long-term temporal and spatial trends within the data. Based on desertification map, we find that 68% of Iran shows a high to very high susceptibility to desertification, representing an area of 1.1 million km^2^ (excluding 0.42 million km^2^ classified as unvegetated). Our results highlight the importance of scale in assessments of desertification, and the value of high-resolution data, in particular. Annually, no significant change is evident within any of the five indices, but significant changes (some positive, some negative) become apparent on a seasonal basis. Some observations follow expectations; for instance, NDVI is strongly associated with cooler, wet spring and summer seasons, and milder winters. Others require more explanation; for instance, vegetation appears decoupled from climatic forcing during autumn. Spatially, too, there is much local and regional variation, which is lost when the data are considered only at the largest nationwide scale. We identify a northwest–southeast belt spanning central Iran, which has experienced significant vegetation decline (2001–2015). We tentatively link this belt of land degradation with intensified agriculture in the hinterlands of Iran’s major cities. The spatial and temporal trends identified with the three vegetation and two climate indices afford a cost-effective framework for the prediction and management of future environmental trends in developing regions at risk of desertification.

## Introduction

Desertification is defined as “land degradation in arid, semi-arid and dry sub-humid areas resulting from climate change and human activities”^1^. Land degradation is emerging as one of the most globally catastrophic issues in the context of contemporary climate change and non-controlled anthropogenic activities^2,3^. Yet, there is no clear consensus among scientists regarding how to combat desertification and land degradation, and the main factors driving these phenomena continue to be debated^4–6^. Here, we consider desertification as the reduction or total loss of land productivity imposed via some combination of soil erosion, degradation of soil properties, and long-term loss of natural vegetation^7,8^. During the last century, approximately 70% of drylands (i.e., semi-arid, arid and hyperarid lands) have manifested signs of desertification and among different land-use categories croplands experience the highest risk, with ~ 70% of the area degraded^9^. Today, more than 250 million people worldwide suffer directly from desertification, while about one billion people in over 100 countries are currently at risk^10,11^. The majority of regions at risk are located in arid and semi-arid areas concentrated within the Global South^12–17^.

The extremely large scale nature of land degradation and desertification means that over recent decades, remote-sensing techniques have been applied widely as tools for evaluating spatial and temporal trends^18–20^. A diverse range of variables and indices extracted from different sensors or satellites, such as AVHRR (Advanced Very-High-Resolution Radiometer), AVHRR-GIMMS (Global Inventory Monitoring and Modelling System), MODIS (Moderate Resolution Imaging Spectroradiometer), NOAA (National Oceanic and Atmospheric Administration) AVHRR or LANDSAT, amongst others, have been employed for assessing land degradation and desertification^11,21–24^. From these sources, large databases have been developed that relate to a plethora of vegetation and climate properties^25,26^, including the normalized difference vegetation index (NDVI)^26–28^, land cover changes^29^, leaf area index (LAI)^30^, land surface temperature (LST)^30^, multidisciplinary indices comprising LAI, albedo and evapotranspiration (ET)^30,31^, water use efficiency (WUE), net primary production (NPP)^32^, enhanced vegetation index (EVI)^33^, and rainfall and vegetation datasets^34^.

Vegetation indices extracted from remote sensing data have been especially useful for monitoring changes in vegetation cover over time. Recent studies have reported the high efficiency of vegetation indices such as NDVI, NPP, LAI and EVI for evaluating the spatial and temporal changes across different scales^35,36^. These variables are commonly correlated with other climate parameters such as rainfall, temperature or evapotranspiration, which are useful for assessing and forecasting the potential for land degradation into the future^24,34–38^. Time-series trends in climate and vegetation indices and the relationships between them have been investigated by many authors^26,38,39^. For example, images from the Tropical Rainfall Measuring Mission (TRMM) sensor have been used to examine the correspondence of vegetation trends with rainfall, while others have studied relationships between land-use changes and land surface temperature (LST)^40^. Evaluating relationships between vegetation dynamics and climate parameters is a proven powerful and efficient means to determine whether imminent climate change and unconstrained human activities pose a threat to food security and sustainable societies in rapidly developing regions of the world^25,26,37^.

Against the above context Iran represents one of the clearest examples of a country deeply affected by land degradation processes such as soil erosion^41,42^, reduction of soil productivity^43–45^ and water quality^46^. Recent modelling studies suggest that major changes in the agricultural and forestry industries present key problems to be solved in the short-to-medium term^47,48^. However, the lack of data noted by several Iranian investigations^49,50^ means that comprehensive nationwide studies that consider recent climate and vegetation trends are scant. With satellite observations now providing long time-series data of relevant parameters at a relatively high spatial resolution (thus overcoming the risk of misinterpreting natural inter-annual variation), a new opportunity arises to explore data at multi-decadal, regional scales, as well as exploring the data with increased granularity to explore temporal and spatial patterning within the data.

## Materials and methods

### Study area

**Figure 1:**
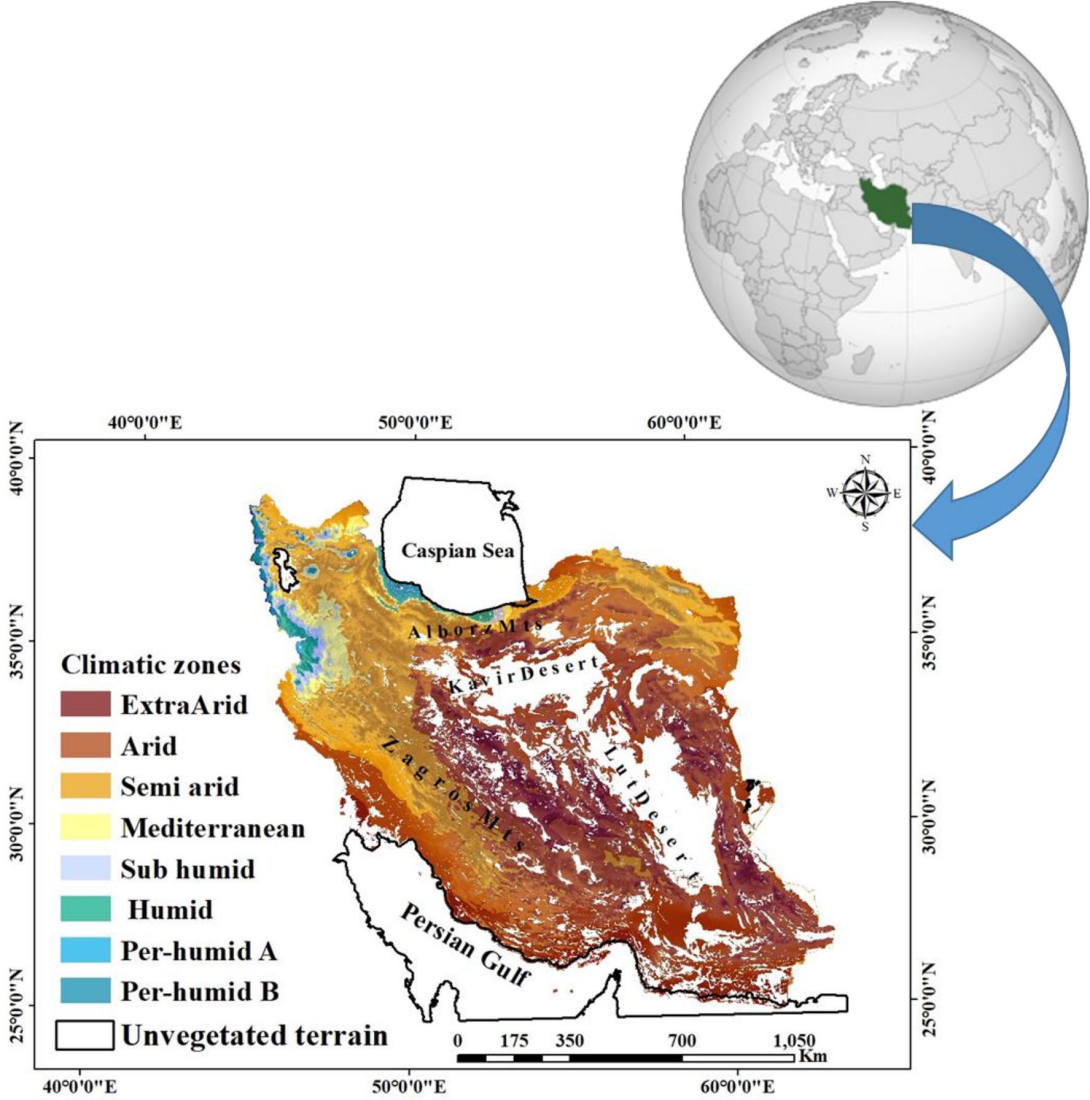
Map of climate zones in Iran based on the Extended De Morton classification (1970–2005)^111^.

Located in southwest Asia, Iran spans an area of pronounced topographic gradients, including elevations of > 5000 m in the Alborz and Zagros mountains, together with coastal areas along the Caspian Sea that are below sea level (Fig. 1). More than 85% of the total 1.6 million km^2^ area of the country is dryland and steppe^51,52^. Regarding rainfall and temperature, Iran spans significant climatic variability. Rainfall averages ~ 2000 mm/yr in the northern and western parts, and ~ 120 mm/yr in central and eastern areas. Temperature extremes can range from − 20 to 50 °C in the southwest and along the northern coast of the Persian Gulf, respectively^53^. These seasonal variations have generated diverse biomes including several endemic vegetation communities^54^. However, high climate variability also makes Iran prone to desertification and land degradation. According to^55^ about 70% of the human population lives in 17 provinces of which 20% are directly affected by desertification^56^. It has been suggested that the amount of the rainfall for Iran may decline 20–25% by 2050^57^. The most factors controlling Iran’s desertification are changing land use, climate changes such as the risk of increasing temperature and decreasing rainfall, increasing population, exploitation of water resources and salinization^58^.

### Databases

Five different indices and parameters, extracted with ArcGIS 10.8 software, were used to conduct a trend analysis of vegetation and climate over the period 2001–2015: (1) NDVI, normalized difference vegetation index; (2) NPP, net primary production; (3) LAI, leaf area index; (4) LST, land surface temperature; and (5) P, precipitation. All data are summarized in Table 1 and the raw data are available at https://neo.sci.gsfc.nasa.gov. We applied simple data pre-processing, such as georeferencing, atmospheric corrections, and projection to WGS-1984 coordinates. Average values of all five indices were calculated for 2001–2015 on a pixel-by-pixel basis. Monthly time series of the three vegetation-specific indices were used for trend analysis of vegetation changes. The monthly time series of the climate indices were adjusted to different resolution from the MODIS and TRMM images, respectively^59–61^. For the trend analysis, first, the values of the five indices were analyzed on seasonal (spring, summer, winter, autumn) and annual timescales. Secondly, correlations between vegetation indices and climate indices were investigated via pixel analysis of the time series. All five indices were then combined into a map format using ArcGIS 10.8 software^62^. We give a brief description of each of the five indices in the text below.

**Table 1:** Summary of datasets used in this study.

Data	Full description	Product	Temporal resolution	Spatial resolution	Data range
NPP	Net primary production	Mod17a3	Monthly	0.1°	0–6 g/m^2^ year
NDVI	Normalized difference vegetation index	MOD13C1	Monthly	0.1°	− 1–1
LAI	Leaf area index	MOD15A2	Monthly	0.25°	0–7 m^2^
LST	Land surface temperature	MOD11C1	Monthly	0.25°	− 25–45 °C
TRMM	Tropical rainfall measuring mission	TRMM	Monthly	0.25°	1–2000 mm

#### Normalized difference vegetation index (NDVI)

MODIS-TERRA monthly NDVI data were examined to quantify variations in vegetation cover at the ground surface (Table 1); this data is commonly used for large spatial scales^25,26,37^. Average NDVI values for each season (spring, summer, winter, and autumn) were calculated; pixels with NDVI > 0 indicate areas with vegetation, pixels with NDVI < 0 show areas without vegetation^63^. Such pixels were excluded from further analysis to decrease the effects of bare ground, snow cover and water.

#### Net primary production (NPP)

The total amount of carbon dioxide produced by plants is commonly termed the NPP and defined as the difference between gross primary production and respiration, which is also called net ecosystem production^64^ (Table 1). NPP has been used previously to study desertification linked to climate change^65,66^. Here, we use MODIS monthly NPP data (2001–2015) following the procedure outlined by^67^.

#### Leaf area index (LAI)

The LAI indicates the activity level of vegetation^68,69^ in terms of the vegetation canopy (%) and the number of leaf layers per unit area^35,70^. MODIS-Terra and -Aqua monthly LAI data were used to derive these parameters (Table 1). The LAI has great potential for modelling global ecosystems, such as simulation of ecological responses to climate change and chemical compounds in the atmosphere^71,72^. The MOD15A2 LAI product used here is a monthly composite tool provided at 0.1 km^2^ spatial resolution. MODIS LAI data was first projected onto an integrated sinusoidal grid then re-projected onto the WGS-84 coordinate system in ArcGIS 10.8. The quality of the data presented in each image dataset is monitored by ascribing ‘low’, ‘marginal’ or ‘good’ quality per pixel.

#### Land surface temperature (LST) index

LST is a suitable index for studying energy exchanges involved in ground-surface processes at different scales^73,74^. Here, we use MOD11C1-TERRA monthly LST data (Table 1). For estimating land surface temperature in these images, a split-window algorithm was applied to optimize the water vapour column and the temperature of lower air layers in the atmosphere^75^. Data pre-processing involved eliminating pixels that have less LST in the case study than in the retrieval errors resulting from surface emissivity uncertainties. Also, cloud-contaminated pixels were eliminated to ensure that only clear sky conditions are assessed, since our focus is on radiative frost.

#### Precipitation (P)

We used the Tropical Rainfall Measuring Mission (TRMM) monthly rainfall data^76,77^ at a spatial resolution of 0.25° and with a monthly temporal resolution (Table 1). This index is generated by merging observations acquired at microwave and infrared radiation wavelengths^78^. Here, monthly TRMM data was used to estimate seasonal and annual precipitation.

### Trend analysis of indices for 2001–2015

We applied the Mann–Kendall non-parametric test and the Theil–Sen estimator to detect temporal variations in all five indices, and the Pearson linear regression coefficient to investigate the correlation between indices. The Mann–Kendall non-parametric test describes the rate of a decreasing or increasing trend between − 1 and + 1, whereby values of + 1, 0 and − 1 denote increase, constant, and decrease, respectively^25^. We also used the z-score whereby an increasing or decreasing trend at 5% significance level is denoted by z ≥ 1.96 and z ≤  − 1.96, respectively^79,80^. Autocorrelation effects in the trend analysis were removed following the approach of^81^. This method evaluates trends H_0_ (negative) and H_1_ (positive) in the data series with a 5% significance level and one-way *p*-value (the probability of random distribution of data). A significant trend is indicated when the *p*-value is < 0.1. The Mann–Kendall method calculates the S statistic^82^, which indicates the sum of the difference between data points, as:

1 $$S = \sum\limits_{i = 1}^{N - 1} {\sum\limits_{j = i + 1}^{N} {{\text{sgn}} \left( {x_{j} - x_{i} } \right)} }$$

where *x*_*i*_ is the observed value at time *j*, *x*_*k*_ is the observed value at time *k*, *j* is the time elapsed since time *k*, and *n* is the duration of the dataset. The sign of the value is defined as:

2 $${\text{sgn}} \left( {x_{j} - x_{i} } \right) = \left\{ {\begin{array}{*{20}l} { + 1,} \hfill & {(x_{j} - x_{i} ) > 0} \hfill \\ {0,} \hfill & {(x_{j} - x_{i} ) = 0} \hfill \\ { - 1,} \hfill & {(x_{j} - x_{i} ) < 0} \hfill \\ \end{array} } \right.$$

When the number of observations is ≥ 10, the statistic S is normally distributed with a mean of 0^79,81^. Therefore, the variance is given as:

3 $$\sigma_{s}^{2} = \frac{1}{18}\left[ {N(N - 1)(2N + 5) - \sum\limits_{i = 1}^{m} {t_{i} (t_{i} - 1)(2t + 5)} } \right]$$

where N is the number of observations and t_i_ is the number of sequences of the sample time series. The statistical significance of *S* is checked using a test statistic or z-score. Test statistic *z* is expressed as:

4 $$z = \left\{ {\begin{array}{*{20}l} {(s - 1)/\sigma_{s} ,} \hfill & \quad {if\;s > 0} \hfill \\ {0,} \hfill & \quad {if\;s = 0} \hfill \\ {(s + 1)/\sigma_{s} ,} \hfill & \quad {if\;s < 0} \hfill \\ \end{array} } \right.$$

where *z* indicates a normal distribution, and *z* > 0 and *z* < 0 show an upward and downward trend, respectively. A useful indicator of the Mann–Kendall test is the Theil–Sen estimator, β, which is the slope of a monotonic trend in the data series. Positive or negative β indicate increasing and decreasing trends, respectively^82–84^. Following^83^, the magnitude of the trend over time is estimated by determining the slope between all possible data pairs and then finding the median value as:

5 $$\upbeta _{{\text{i}}} = {\text{median }}\left( {\frac{{{\text{X}}_{{\text{i}}} - {\text{X}}_{{\text{j}}} }}{{{\text{i}} - {\text{j}}}}} \right)\;\upbeta _{{\text{i}}} = {\text{median }}\left( {\frac{{{\text{X}}_{{\text{i}}} - {\text{X}}_{{\text{j}}} }}{{{\text{i}} - {\text{j}}}}} \right)$$

where i = 1,2 … *N* and *x*_*i*_ is data measurement at time *i*, *x*_*j*_ is data measurement at time *j*, *i* > *j*. For *n* values of the time series of *x* results, N = n(n − 1)/2 values of $\upbeta \upbeta _{{_{{\text{i}}} }}$ ^83,84^. The Pearson correlation coefficient (*r*) was used to indicate a positive correlation (+1), negative correlation (−1), or the absence of correlation (0) between our indices^85^.

A linear regression method^25^ is applied to analyze temporal trends in the NPP, NDVI, LAI, LST and P observations. To obtain the linear regressions, we modelled the series of annual NPP, NDVI, LAI, LST and P values per pixel using the Earth Trends Modeler of the Terrset 2020 software^25^:

6 $$R_{x \cdot y} = \frac{{\mathop \sum \nolimits_{i = 1}^{n} \left( {x_{i} - X} \right)\left( {y_{i} - y} \right)}}{{\sqrt {\mathop \sum \nolimits_{i = 1}^{n} \left( {x_{i} - x} \right)^{2} \times \mathop \sum \nolimits_{i = 1}^{n} (y_{i} - y)^{2} } }}$$

where ${\text{R}}_{{{\text{x}} \cdot {\text{y}}}}$ indicates the correlation coefficient, and x_i_ and y_i_ are the dependent and independent variables, respectively.

### Spatial patterns of land desertification

The five indices were used to identify spatial patterns in desertification potential. Although each index can describe an aspect of desertification, it is more useful to integrate multiple indices^86^, as we have done here. Using a Boolean classifying method and a re-classification technique, each of the five indices was subdivided into five classes, indicating very low, low, moderate, high, and very high potential for desertification (Table 2). For example, if the Boolean approach combines the lowest 20% of pixels among NDVI, NPP, LAI, and P, with the highest 20% of LST values, the area can be classified as having very high (maximum) desertification potential (Table 2). The five classes were mapped using ArcGIS 10.8^66,86^.

**Table 2:** Land desertification classes based on the Boolean classification method for normalized difference vegetation index (NDVI), net primary production (NPP), leaf area index (LAI), land surface temperature (LST), and precipitation (P).

Class no	NDVI (%)	NPP (%)	LAI (%)	LST (%)	P (%)	Desertification class
1	80–100	80–100	80–100	0–20	80–100	Very low
2	60–80	60–80	60–80	20–40	60–80	Low
3	40–60	40–60	40–60	40–60	40–60	Moderate
4	20–40	20–40	20–40	60–80	20–40	High
5	0–20	0–20	0–20	80–100	0–20	Very high

## Results

### Variations in vegetation and climate indices over time

**Figure 2:**
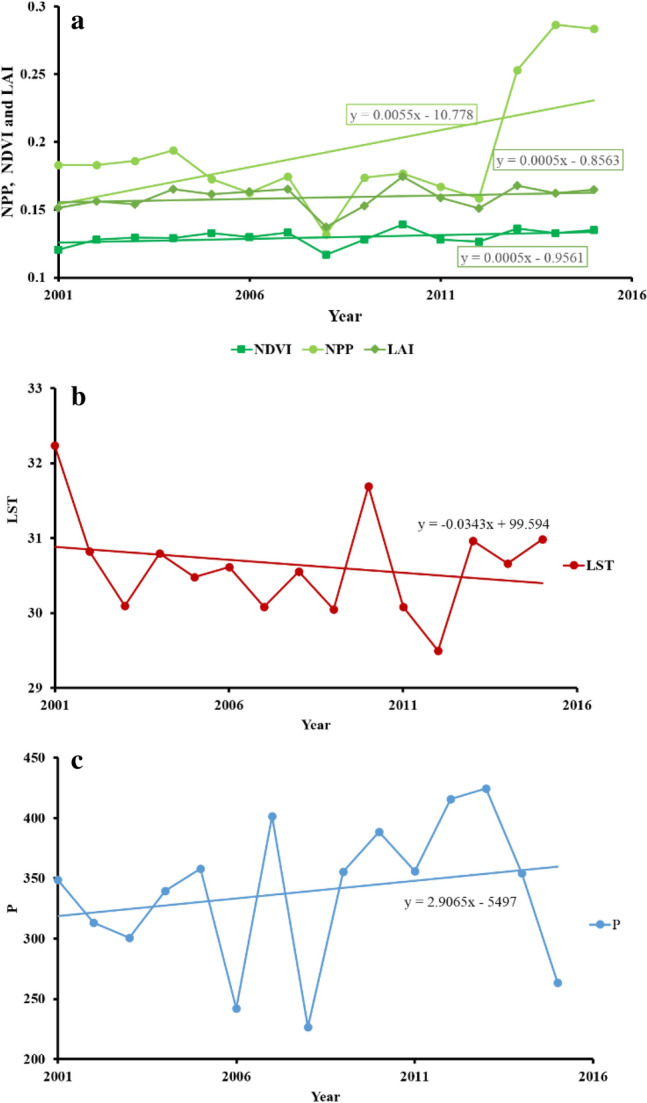
The average of annual variations (2001–2015): (**a**) NDVI, NPP and LAI, (**b**) LST and (**c**) P. Linear regressions indicate possible trends over time.

In terms of annual variations, no statistically significant trends emerged during the period 2001–2015 (Fig. 2 and Table 3). However, according to the Mann–Kendall τ statistic and z statistic, and the β statistic (slope) of the Theil–Sen estimator, the trend was non-significant positive for NDVI, NPP, LAI and P, while the trend direction for LST was non-significant negative. Several of the indices, most notably NDVI and LAI show a marked decline in 2008, and both 2006 and 2008 are characterized by negative precipitation anomalies of around 30%.

**Table 3:** Statistical significance of the trend lines shown in Figs. 2 and 3.

Series\test	Kendall's tau	*p*-value	Sen's slope	Series\test	Kendall's tau	*p*-value	Sen's slope
**Annual data**				**Seasonal NDVI**			
NDVI	0.314	0.113	0.001	NDVI (Spring)	0.295	0.138	0.001
NPP	0.077	0.729	0.001	NDVI (Summer)	− 0.105	0.621	0.000
LAI	0.219	0.276	0.001	NDVI (Autumn)	**0.638**	**0.001**	0.000
LST	− 0.105	0.621	− 0.015	NDVI (Winter)	0.314	0.113	0.001
P	0.219	0.276	3.799				
**Seasonal P**				**Seasonal NPP**			
P (Spring)	− 0.249	0.215	− 2.457	NPP (Spring)	0.077	0.729	0.001
P (Summer)	0.115	0.586	0.271	NPP (Summer)	0.096	0.656	0.002
P (Autumn)	**0.478**	** < 0.0001**	3.187	NPP (Autumn)	0.268	0.181	0.003
P (Winter)	− 0.383	0.053	− 3.621	NPP (Winter)	0.345	0.083	0.003
**Seasonal LST**				**Seasonal LAI**			
LST (Spring)	0.010	1.000	0.008	LAI (Spring)	0.200	0.322	0.001
LST (Summer)	0.143	0.488	0.019	LAI (Summer)	0.010	1.000	0.000
LST (Autumn)	− 0.486	1.000	− 0.095	LAI (Autumn)	0.067	0.767	0.000
LST (Winter)	0.124	0.553	0.054	LAI (Winter)	0.162	0.428	0.001

Bold values indicate statistically significant with α < 0.01.

Note that based on annual data, no index or climate parameter shows a significant trend over the period studied (2001–2015). Once dissociated to a monthly level, only the autumn period shows significant trends, in both increased rainfall and increased NDVI.

**Figure 3:**
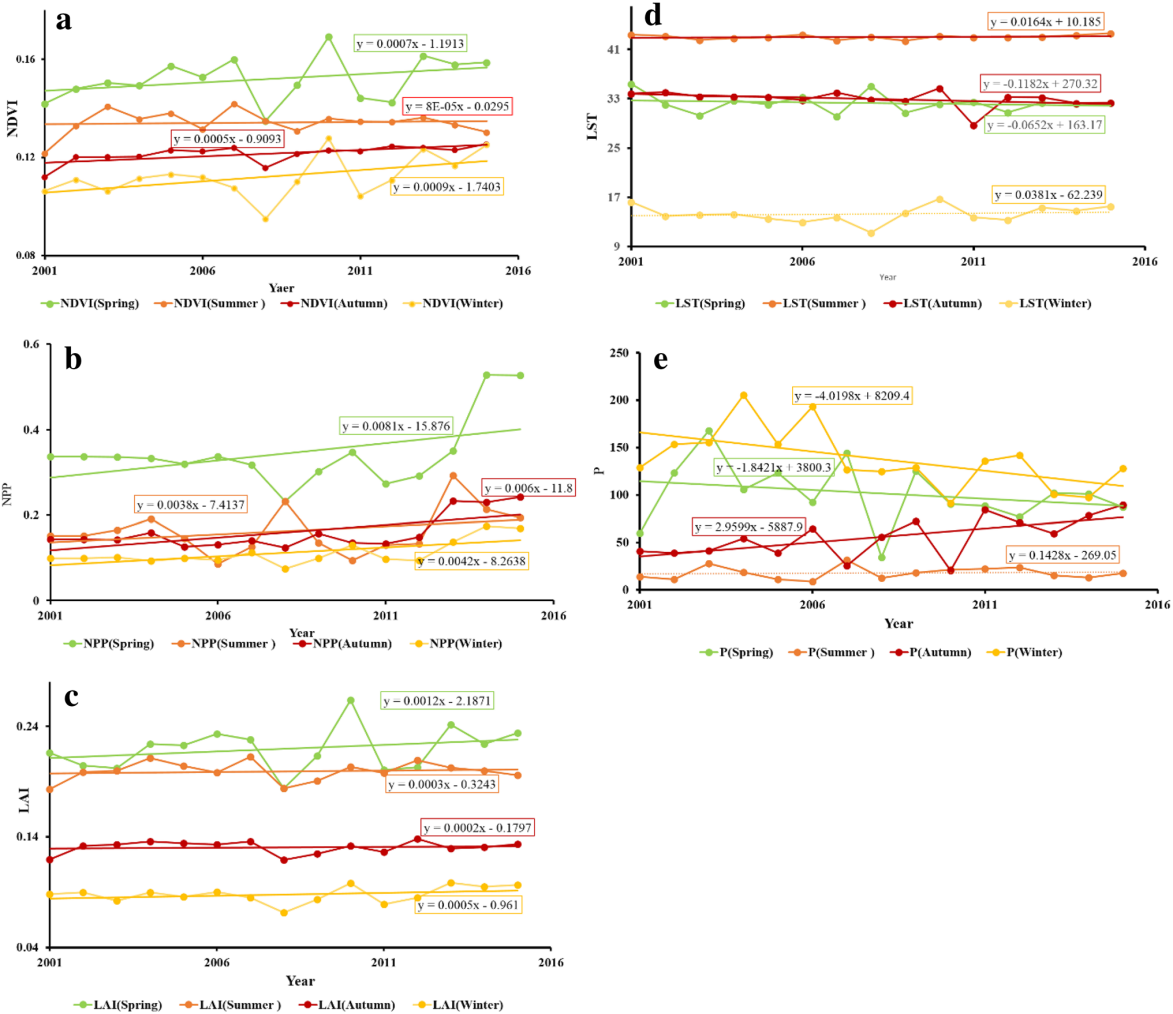
Seasonal (spring, summer, autumn, winter; 2001–2015) trends in (**a**) NDVI, (**b**) NPP, (**c**) LAI, (**d**) LST and (**e**) P.

Dissociating the trends by season, however, yields some limited, but highly significant, evidence of change over the study period. Seasonal variations in NDVI, NPP, LAI, LST, and P are shown in Fig. 3 and Table 3.

Although very few of the relationships explored here show a significant trend, two stand out as highly significant (> 99.9% confidence). Autumn precipitation has increased during the interval, as has the autumn NDVI. It is worth noting that the winter precipitation (P) trend during the period 2001–2015 however, is negative, and lies only just outside significance at 95% confidence; spring and summer are non-significant declines. All other vegetation indices by season are non-significant positive trends, and climatic parameters are more varied. All seasonal changes in temperature (LST) are non-significant increases.

Some visual patterns within the data are not picked up as readily by slope analysis of the time series. Notable amongst these are marked drops in precipitation, in spring 2008 and autumn/winter 2010, which are accompanied by all-season dips in NDVI for 2008. Spring 2010 shows a marked peak in activity in all vegetation indices, and more surprisingly, given the autumn/winter decline in rainfall, a lesser peak is evident for winter 2010, especially for the NDVI and LAI indices.

The rationale for correlation varies depending on the paired statistics. Firstly, the degree of correlation between the different vegetation indices (NDVI, NPP and LAI) provides some indication of their robustness, although the scope for autocorrelation must be noted^87^. Secondly, the correlation between climate parameters (LST and P) helps unpick the nature of climatic forcing. Lastly, correlations (or, equally importantly, lack of) between vegetation indices and climate parameters may distinguish natural and anthropogenic drivers of landscape change. The different relationships are shaded differently in Tables 4 and 5 to facilitate this interpretation.

**Table 4:**
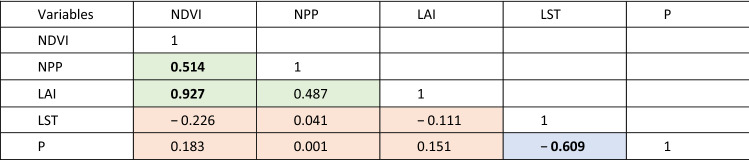
Correlation (*r*-value) between the vegetation and climate indices using the annual time series (Fig. 2).

Correlations between vegetation indices are shaded green, and are used to explore the relative merits of the vegetation products employed here. Correlations between the climatic indicators are shown in blue, and are used to explore the relationship between precipitation and temperature. Correlations between climatic indices and climate parameters are shaded orange, and are used to explore the strength of relationships between climatic forcings and vegetation response. Values in bold differ from 0 with a significance level α < 0.05.

**Table 5:**
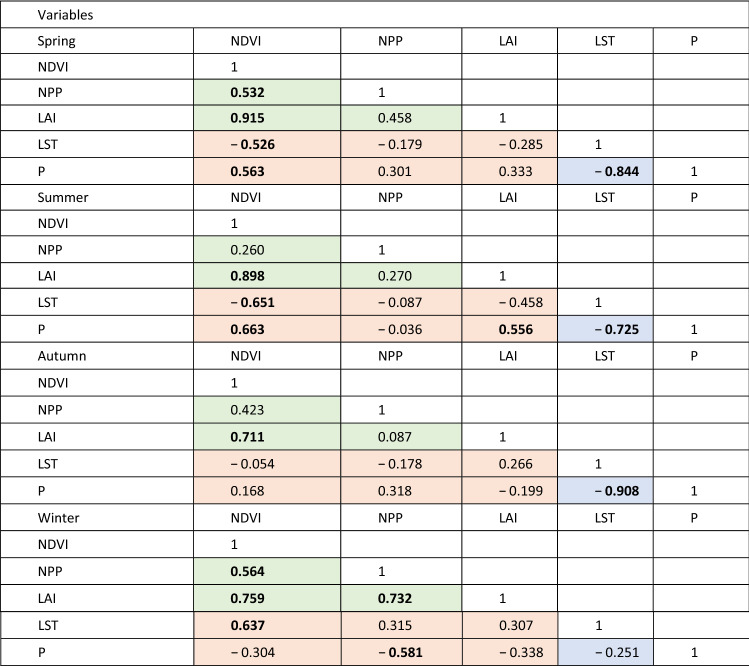
Correlation (*r*-value) between the vegetation and climate indices using the seasonal time series (Fig. 3).

Correlations between vegetation indices are shaded green, and are used to explore the relative merits of the vegetation products employed here. Correlations between the climatic indicators are shown in blue, and are used to explore the relationship between precipitation and temperature. Correlations between climatic indices and climate parameters are shaded orange, and are used to explore the strength of relationships between climatic forcings and vegetation response. Values in bold differ from 0 with a significance level α < 0.05.

Correlations between NDVI, NPP, LAI, LST, and P over time are summarized for the annual time series in Table 4. The different vegetation indices correlate positively, with NDVI and LAI correlating very strongly, and significantly. The correlation between NDVI and NPP is weaker but still significant at α < 0.05, and that between LAI and NPP positive, yet below the threshold of significance. Similarly, the two climatic parameters, as might be expected, correlate inversely; hotter years see less rainfall overall. However, no significant correlations are observed at all between the climatic and vegetation parameters.

The seasonal correlations of the five indices (Table 5) reveal a much more nuanced set of relationships. The reason for the lack of correlation between some of the vegetation and climatic parameters in this environment becomes clear. The inverse relationship between temperature and precipitation remains strong year-round, but is at its weakest in winter (when, indeed, it is not significant at 95% confidence). The relationships between the different vegetation indices vary substantially throughout the year. In winter, all three relationships are strongly correlated, but in spring this drops to two (NDVI vs NPP and NDVI vs LAI), and in summer and autumn, only NDVI and LAI remain correlated; NPP becomes entirely dissociated with the other vegetation indices. When considering the relationships (or apparent lack thereof) in the correlations between climatic and vegetation parameters, the role of seasonality in the generally hot, arid climate of Iran becomes clear. NDVI remains the most closely linked of the climatic parameters to the vegetation response, and is correlated negatively with spring and summer temperatures, and positively with rainfall in these seasons. In autumn there is no significant relationship between NDVI (or any other vegetation index) and either of the climatic parameters, and in winter, the relationships invert; a higher NDVI index is now associated with warmer temperatures and less precipitation (albeit not significantly in the latter case). The trends for the other vegetation indices (NPP and LAI) generally mirror those of NDVI, albeit typically at lower significances, and are generally insignificant.

### Spatial variation in vegetation and climate indices

**Figure 4:**
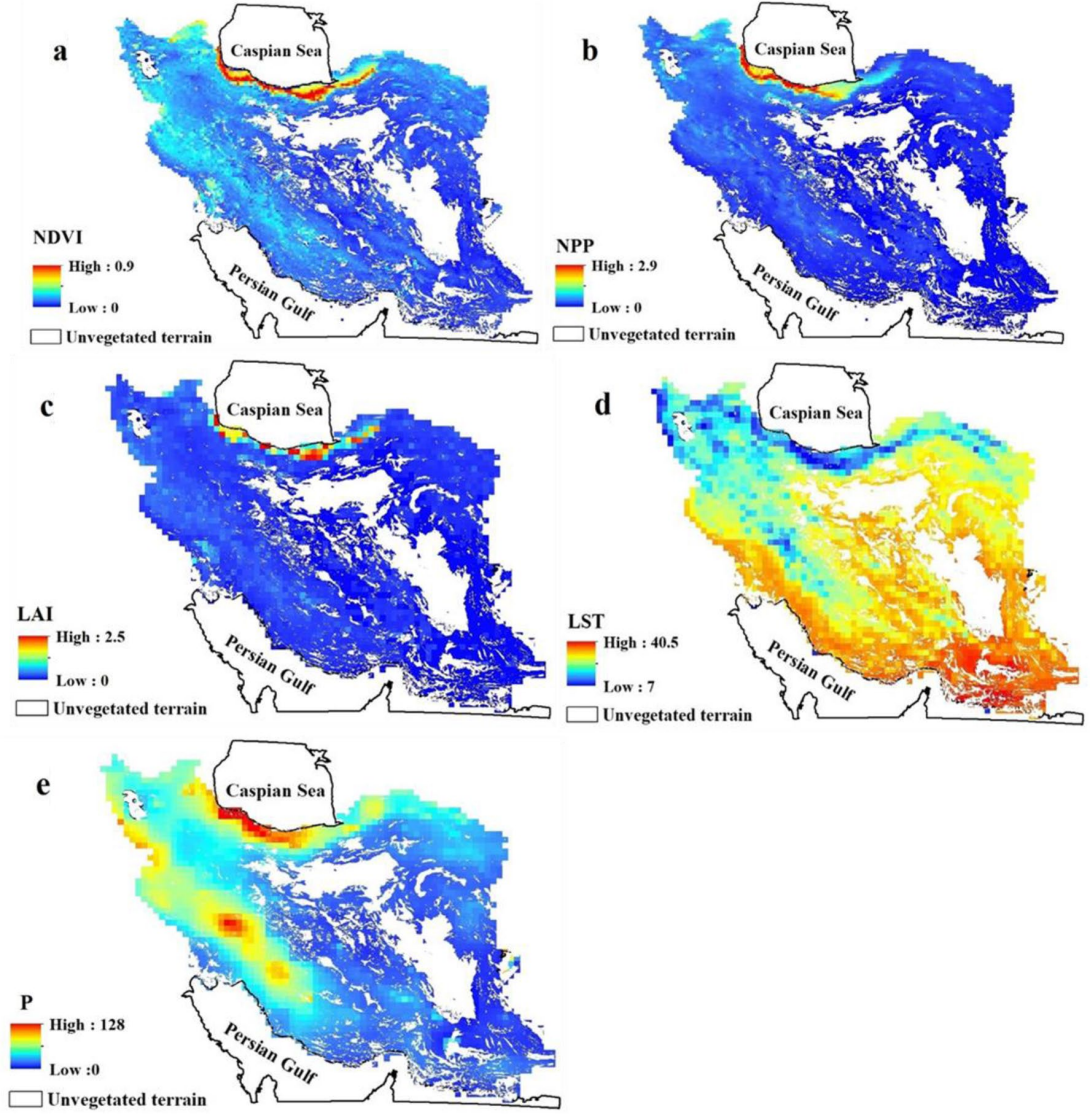
Maps of Iran showing the mean spatial variation of (**a**) NDVI, normalized difference vegetation index; (**b**) NPP, net primary production; (**c**) LAI, leaf area index; (**d**) LST, land surface temperature; and (**e**) P, precipitation for the period 2001–2015. Unvegetated areas due to hyperaridity are excluded from our analysis and shown blank (e.g. the Lut desert, Dasht-e-Kavir, some dunes and other desert surfaces). All maps prepared in ArcGIS 10.4.1 (https://www.esri.com/en-us/about/about-esri/overview).

Figure 4 shows the mean values for NDVI, NPP, LAI, LST, and P for the period 2001–2015, and reveals substantial spatial variability. All vegetation indices reveal high values only in the regions bordering the Caspian Sea, which is significant as it is the only region of Iran with agricultural land rated as ‘very good’^88^. NDVI (Fig. 4a) also highlights regions of the Zagros Mountains and the Khuzestan Plain (forming the northern end of the Persian Gulf) as having moderate vegetation health; these regions are shown less clearly in the NPP and LAI (Fig. 4b, c) indices, however. LST is, unsurprisingly, very high throughout much of Iran throughout the study period (Fig. 4d), with only the western portion of the Zagros Mountains and the Caspian coast benefiting from more moderate temperatures. Much of the south and southwest of the country—even in regions not excluded from analysis due to very low NDVI scores—the long-term average land surface temperature has been in excess of 30 °C, or even 40 °C in the far southwest. Precipitation follows a near-inverse spatial pattern to temperature, with the Caspian coast and Zagros Mountains the only regions with substantial precipitation; only the far northwest and far northeast of the country exhibit this trend to a limited degree, being very dry but cooler.

**Figure 5:**
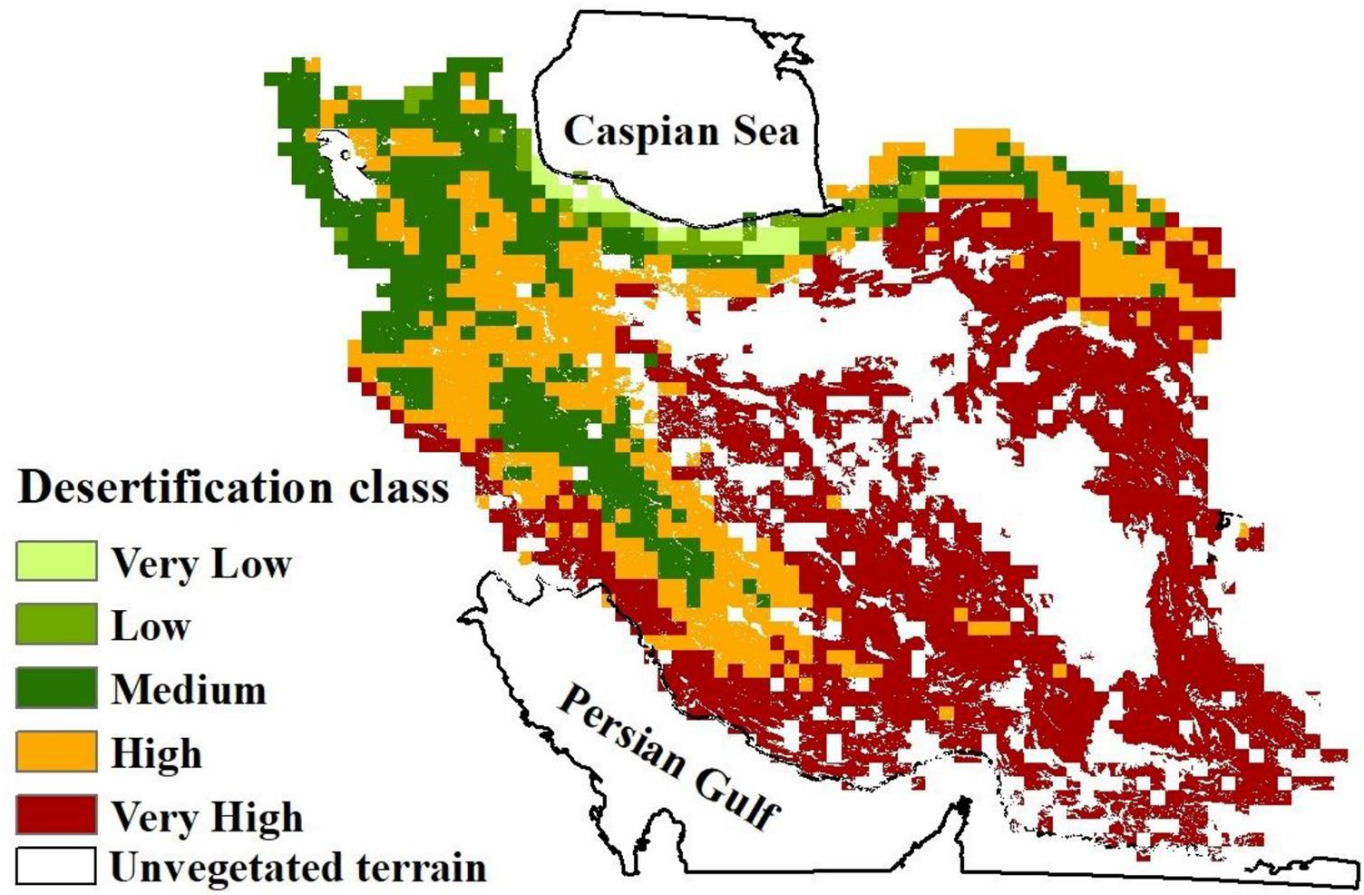
Map of Iran showing areas susceptible to desertification during the period 2001–2015. Unvegetated areas due to hyperaridity are excluded from our analysis and shown blank. Map prepared in ArcGIS 10.4.1 (https://www.esri.com/en-us/about/about-esri/overview).

Based on our five vegetation-climate indices, we find that just over 80% of Iran shows a high to very high susceptibility to desertification (Table 6). When all five indices are combined, the net results suggest high to very high potential sensitivity to desertification across 68% of Iran (Table 7 and Fig. 5). Considering the total land area of Iran (excluding non-vegetated terrain), susceptibility to degradation is estimated at 41.4% very high, 26.2% high, 5.5% medium, and 1.4% low or very low (Fig. 5 and Table 7).

**Table 6:** Proportion (%) of vegetated land classified as being at different risks of desertification over the period 2001–2015 based on NDVI, NPP, LAI, LST, and P.

Desertification risk	NDVI (%)	NPP (%)	LAI (%)	LST (%)	P (%)
Very Low	1	0.7	0.5	0.1	1.2
Low	1.2	0.3	0.8	2.0	0.2
Medium	2.1	1.0	0.7	17.7	10.0
High	29.5	2.8	2.3	39.7	34.8
Very High	66.2	95.2	95.7	40.5	53.8
Total	100	100	100	100	100

**Table 7:** Summary land degradation susceptibility for Iran over the period 2001–2015.

Class	Pixel count	Area (million km^2^)	Area (%)
Very low	5	0.003	0.18
Low	33	0.02	1.22
Medium	144	0.09	5.48
High	684	0.43	26.17
Very high	1092	0.68	41.39
Presently unvegetated terrain	670	0.42	25.56
Total	2628	1.64	100

### Spatio-temporal trends

**Figure 6:**
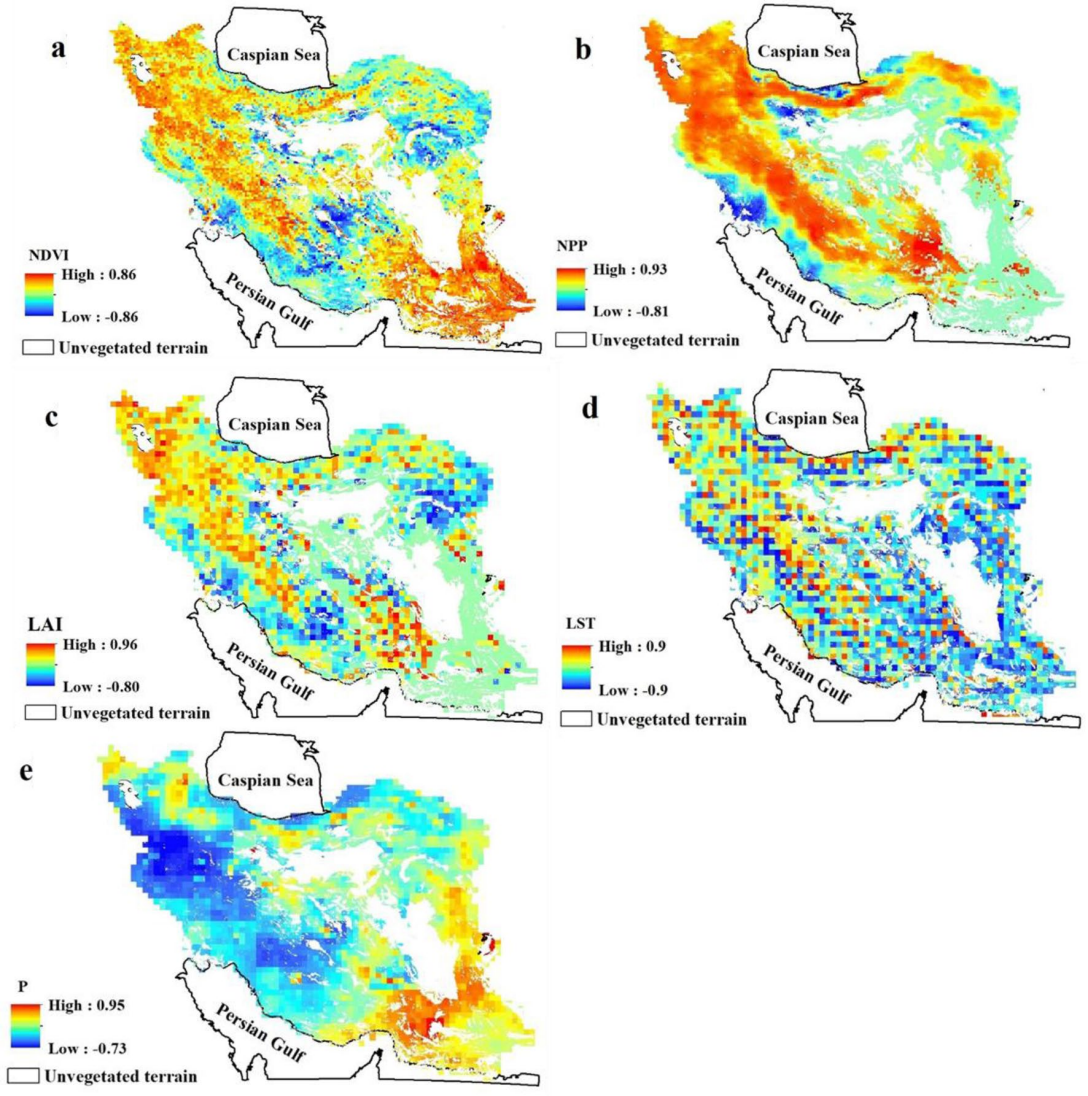
Maps of Iran showing the *r*-value derived from the linear regression trends and analysis of annual observations: (**a**) NDVI, normalized difference vegetation index; (**b**) NPP, net primary production; (**c**) LAI, leaf area index; (**d**) LST, land surface temperature; and (**e**) P, precipitation during the period 2001–2015. Unvegetated areas due to hyperaridity are excluded from our analysis and shown blank (e.g. the Lut desert, Dasht-e-Kavir, dunes, and other desert surfaces). All maps prepared in ArcGIS 10.4.1 (https://www.esri.com/en-us/about/about-esri/overview).

By analyzing the temporal change in the data on a pixel-by-pixel basis, spatio-temporal trends in the data can be further explored. Figure 6 shows the *r*-values obtained from linear regressions applied to annual observations of the five climate and vegetation indices for the period 2001–2015. Here, much spatial variation is evident, and the *r*-values for each of the five indices vary much more markedly than when considered as a region. Indeed, for each of the five indices, when considered on a pixel-by-pixel basis, the temporal trends during the years 2001–2015 range from strong negative correlations (minimum values range from *r* =  − 0.73 to *r* =  − 0.9), and strong positive correlations (maximum values range from *r* = 0.86 to *r* = 0.9). Whilst a much wider range of values is to be expected given the very large number of correlations being considered here (i.e. it is more likely that some strong correlations might occur by chance), strong spatial coherence and autocorrelation in the data here suggest that this is driven by localized factors in the environment.

For the vegetation indices, a fairly consistent spatio-temporal pattern emerges, although there are subtle differences between the indices. NDVI (Fig. 6a) shows an increasing trend through the northwest-southeast trending Zagros Mountain region, across much of the Caspian coast and through into the driest part of the country, the hyperarid southeast. However, a northeast–southwest belt across the center of Iran has experienced the opposite trend in NDVI during this interval; that is, decreasing vegetative health. This includes a range of topographies, from the mixed topography of the far northwest, the southern reaches of the Zagros Mountains, and across to the Khuzestan Plain (the norther end of the Persian Gulf), previously identified by NDVI as being one of Iran’s more productive lands. The pattern for NPP (Fig. 6b) is similar, and perhaps spatially more coherent. Similar regions are identified as experiencing positive trends in vegetation productivity during the interval 2001–2015, and a similar northeast-southwest belt of declining productivity across the center of Iran. More minor differences exist; there is markedly less evidence from the NPP index for positive trends in the arid southeast, and localized regions of the Caspian coastal region are highlighted as showing strong negative trends over this timeframe. The LAI data (Fig. 6c) show a very similar pattern to the NPP index.

In terms of the climatic parameters, again, some spatio-temporal coherence emerges, although this is a good deal noisier in the case of the LST data. Very broadly (Fig. 6d), the western half of Iran has tended to experience an increasing temperature over the period, and the hyperarid east has seen temperatures generally decrease. However, there is much localized variability; some of it seemingly spatially coherent and likely indicating local/regional variance (e.g. a narrow belt of decreasing temperatures to the south of the Zagros, and small patches of decreasing temperatures around the Caspian), and some of it (region-wide) much less spatially coherent and likely the results of noisy climate input data. Precipitation trends are much more well-defined spatially. Again, an east–west divergence is apparent; broadly speaking, the hyperarid east has received an increasing trend in rainfall during the interval 2001–2015, and the majority of the rest of the country has seen a decline in rainfall during this interval (with the exception of a small region in the far northwest).

Overall, once spatially disaggregated, the temporal trends suggest more concerning patterns for significant regions of Iran. The variations of NPP, NDVI and LAI indicate a significant decreasing trend in 65%, 69% and 47% total area respectively, whereas the trend of P declines and LST shows an increasing trend for 60% and 70% total area respectively.

## Discussion

Dryland regions of the world are susceptible to degradation and desertification, and numerous examples^87,88^ illustrate the effects of long-term vegetation loss over time, including an increase in overland flow and associated loss of fertile topsoils. Land degradation poses a serious threat to the sustainable development of growing economies and may even undermine their political stability. Here, we have applied a set of quantitative methods with the aim of conducting a nationwide assessment of Iran’s susceptibility to desertification over the period 2001–2015. We have structured our analysis in terms of the temporal and spatial variations in the five climate-vegetation indices across Iran, at different scales, and the correlation of our climate and vegetation indices.

### Temporal trends in climate and vegetation between 2001 and 2015

Analysis of the country-wide annually-averaged climate and vegetation indices reveal no significant (at 95% confidence) regionally consistent trend in any of the five indices studied (Fig. 2 and Table 3). Initially, this may seem to contradict studies which have suggested that not only land-use change but also spatial and temporal variations in P and LST contribute to land degradation and desertification in arid and semi-arid areas. For instance, a 27-year study of the Zayandehrud basin in Iran reported that variations in temperature and rainfall are significantly impacting on land-use changes as well as changes in land surface temperature^55^. Likewise, compelling evidence has been put forward^89^ that over longer time-series, temperatures in Iran are indeed increasing, as would be expected under the impact of anthropogenic climate change. However, these observations are not necessarily contradictory, and while long-term trends may not be evident in these time-series’ at annual resolution, there are certainly patterns within the data; for instance, the impact of drought years in 2008 (well-reported elsewhere; e.g.^63,65^) is clear in the both the climatic and vegetation series (Fig. 2). Such short-lived excursions in both climatic parameters and vegetation response are very typical of natural dryland environments, however, and should not be taken in isolation and conflated with evidence for long-term degradation. Such short-lived events in vegetation health (using NDVI, NPP and LAI) as a result of drought have been observed elsewhere, both in other region and in global syntheses^90–92^.

A further key issue here is one of scale; both temporal and spatial. To this end, we disaggregated the data seasonally (Fig. 3 and Table 3). Although highly significant trends were identified in the regional data, they indicate a highly significant (> 99.9% confidence) increase in precipitation in autumn (only), and presumably resultant increase in NDVI in the same season. Importantly for land degradation, autumn rains may not be the most important. While they will aid in groundwater recharge, and may be more effective at doing so due to lower land surface temperatures, rains immediately preceding, and during, the growing seasons are likely to be more significant. Evidence for this can be found in Fig. 3c. The aforementioned drought in 2008 is most clearly shown at a seasonal level, with the near-complete failure of the spring rains. Short-term excursions are also evident in the LST record, which registered a decreasing trend in winter, summer, and spring between 2001 and 2004 with temperature reductions of 2.7 °C, 4.4 °C, and 1.3 °C, respectively. From 2004 to 2015, temperature increases in winter, autumn and spring were 0.6 °C, 0.7 °C and 1.9 °C, respectively. Such variations are consistent with the results reported by^89^, who evaluated temperature and precipitation in Iran during the period 1987–2010.

### Correlations between vegetation and climate indices

The relationships between different climatic parameters and vegetation indices (Tables 4 and 5), as noted in the methodologies, can be interpreted in different ways depending on the pairs of variables chosen. They are also of different direct relevance to the aims of this study. For instance, we correlate P and LST (shown in blue on Tables 4 and 5), which reveal a significant negative correlation both in the annual data (Table 3) and three of the four seasons (spring, summer and autumn), with only winter correlation non-significant and negative. However, this largely confirms expected and obvious relationships; precipitation requires cloud cover, which lowers land surface temperature. The reason for the apparent dissociation between these variables in winter is currently unclear.

Correlations between different vegetation parameters (shown in green on Tables 4 and 5) offer some indication of confidence in the different indices, although care must be taken with these interpretations. Firstly, these indices are not necessarily independent in their derivation, and thus the meaning of correlation may be questionable^93^. Secondly, correlation (or lack thereof) raises the simple question as to which is more meaningful for the aims of this study, and without ground verification, this question is ultimately unanswerable within the scope of this study. Nonetheless, we suggest that consistency (or otherwise) of correlations may provide some information on the utility of the indices. Within the annual data (Table 4), NDVI correlates positively and significantly with both NPP and LAI annually, and in six out of eight seasonal comparisons (Table 5). NPP and LAI, conversely, correlate poorly on the whole; the annual relationship is non-significant, as it is for three of the four seasons (winter being the exception, when a strong correlation is observed). We suggest that, due its stronger central position in the correlation matrix, NDVI may be the preferred vegetation index for interpretation.

The correlations between climatic parameters and vegetation indices (shown in orange on Tables 4 and 5) are likely to offer most insight directly related to the aims of this study. Not only might they offer evidence of naturally-induced environmental landscape change, they may help to dissociate the impacts of temperature and rainfall, and equally important, suggest where factors other than climate (e.g. anthropogenic forcing) are impacting vegetation.

Similar to the temporal trend data, at annual resolution, no significant correlations are observed between any of the vegetation indices and either of the climatic parameters (Table 4). Given the lack of trend evident at annual resolution, however, it is difficult to read this as straightforward anthropogenic forcing on landscape change. Seasonally, however (Table 5), significant (> 95% confidence) correlations become apparent. NDVI correlates most frequently and (unsurprisingly for a dryland region) increased NDVI is associated with cooler spring and summer temperatures, warmer winters, and increased rainfall in spring and summer. NPP and LAI generally follow the same direction of correlation as NDVI, but are less frequently significant, although NPP does yield a significant relationship with decreases in winter rainfall; for NDVI, this inverse relationship was suggested, but not significantly so. This seems to indicate that, in winter, temperature rather than rainfall, is the limiting factor for vegetation growth. We suggest that future studies should focus on the detection of specific factors that directly affect temperatures, such as urban-industrial areas, topography, and wind^94,95^. None of the indices correlate with any of the vegetation parameters during autumn. The reason for this is unclear, but may relate to harvesting of crops during this season.

Another factor to be considered is elevation. In Iran, in the Hamadan province, using average rainfall data from 35 synoptic stations spanning 30-years of measurements^96^, studied the impacts of elevation on rainfall distribution. They reported that elevation exerts a control of rainfall. Changes in rainfall are among the main concerns associated with potential climate change effects—as others have suggested in the context of soil erosion linked to extreme weather events in Mediterranean areas^97,98^. Similar outcomes emerge in our new results for Iran where the seasonal variation of the P index exhibited increases of 13 mm and 42 mm in autumn 2001 and spring 2004, respectively. However, from 2004 to 2015, a reduction in rainfall amounting to 19.4 mm, 1.5 mm and 4.9 mm, were observed in winter, autumn and spring, respectively, confirming the results of^89,99,100^. Over our study period, there was a significant drop in precipitation in all seasons between 2008 and 2009.

### Desertification risk mapping

Based on the results of our pixel-based analysis of vegetation indices > 70% of Iran is characterized by sparse vegetation development; mostly in central, eastern, southern, southwestern and, to some extent, northwestern regions of the country. In terms of the climate factors LST and P, these areas are characterized by the highest temperatures and the lowest rainfall. Our new results therefore broadly agree with those of^101^ who reported that > 80% of the land area of Iran occurs in arid and semi-arid regions, in which the vegetation is limited by high temperatures and low rainfall. Only ~ 2% of the area of Iran is rich in vegetation cover, where the climate is naturally favourable. Characterized by a Mediterranean-type humid to very humid climate, these regions mostly occur in northern areas of the Alborz Mountain range, the areas near the Caspian coast and western areas limited to the Zagros Mountains.

Our new spatial mapping of land degradation and desertification in Iran suggests a correlation between the risk of desertification, and the initial suitability of land for agriculture identified by other studies. For example^102^, studied the suitability of lands for agriculture using high-resolution data in Iran and reported that > 80% of the country is unsuitable for agriculture mainly due to rainfall deficiency. The same work concluded that ~ 50% of land under agriculture is not of adequate quality for sustainable production. In a study in the central regions of Iran^103^, it has been reported that only 9.4% of their study area exhibited a low level of desertification risk, whereas > 90% was classified as being at moderate to very high desertification risk.

### Spatio-temporal patterning

It has been shown that for the time-series data and index correlations increased granularity (considering seasonal, rather than annual, timescales) is important in discovering relationships within these data. Figure 6 illustrates that the same is true, most probably more-so, spatially. Although country-wide, temporal trends over the period 2001–2015 were insignificant, Fig. 6 reveals highly significant localized trends in the data during this period; some positively and some negatively correlated. For most indices, even if trends may be below a significance threshold, the high degree of spatial autocorrelation (LST being something of an exception) suggests that these trends are genuine. It also implies that when averaged at a nationwide level, spatial variation in these trends tends to average out, and important information is lost.

For the climatic forcings, although noisy, Fig. 6d suggests a general cooling of the hyper-arid far east of Iran, and a warming of the west. Precipitation (Fig. 6e) suggests a slightly more complex pattern, with the hyper-arid far southeast generally experiencing an increase in rainfall, along with the temperate far northwest, but an extensive belt across much of the center of Iran receiving less rainfall at the end of the study window.

The vegetation indices (Fig. 6a–c) reveal a broadly coherent picture, albeit with some variations between the indices. In each, a positive trend in vegetation during the period 2001–2015 is observed for the northwest of the country (the region with the most temperate climate presently), and a stationary (NPP- and LAI-derived) or increasing (NDVI-derived) trend in vegetation for the far southeast (presently the most arid region of the country). These are both regions where (Fig. 6e) precipitation has increased significantly in some areas during this interval. Across a broad swathe of central Iran, however, stretching from the northwest to the Persian Gulf coast, there is a belt of significantly decreased vegetation during this period (blue shading on Fig. 6a–c). Whilst there is some coherence here with locations experiencing increased rainfall during this interval, there are also many regions where the climatic forcings of P and LST appear detached from the resultant vegetation decline.

### Desertification in Iran: synthesis and policy implications

**Figure 7:**
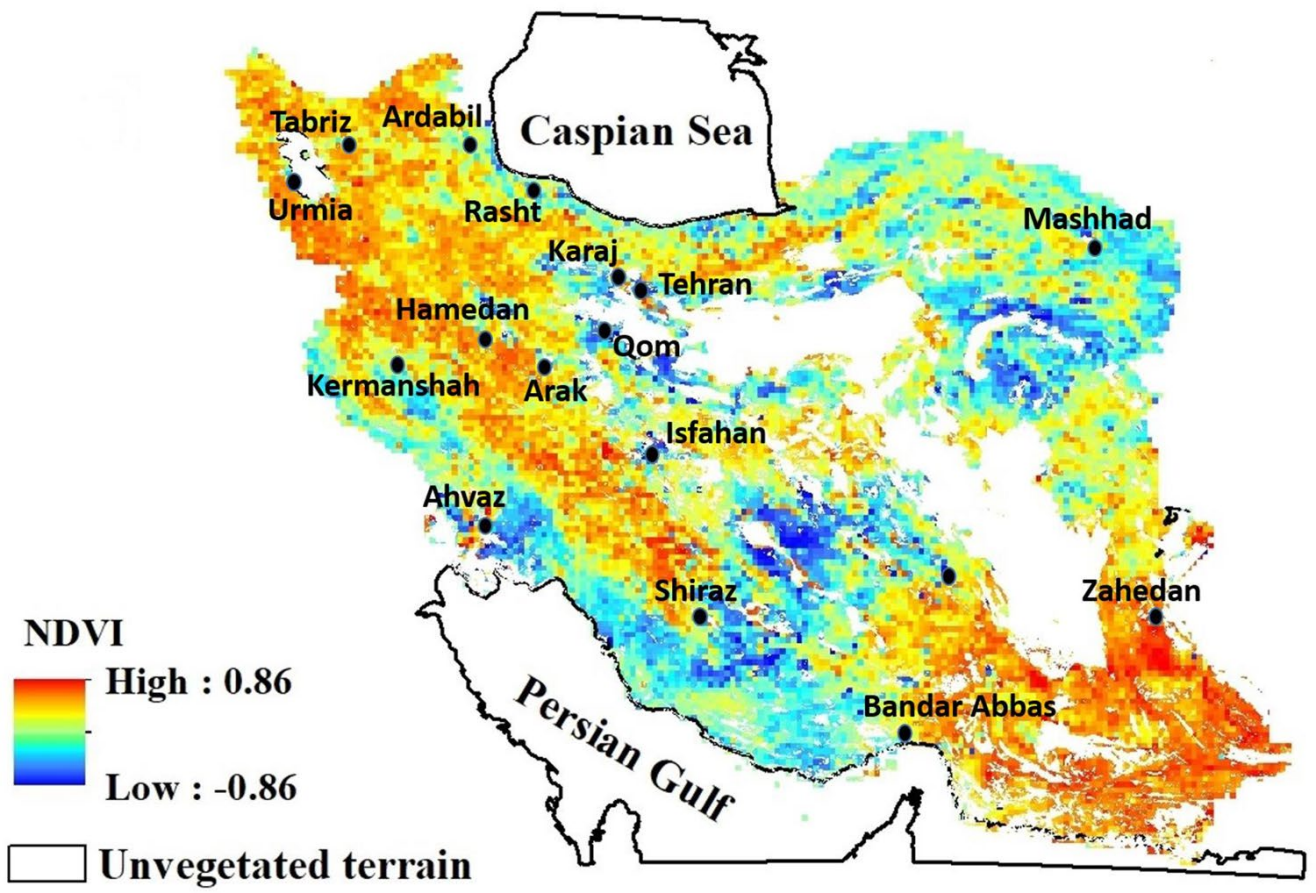
The vegetation trend from 2001 to 2015, as identified using the NDVI, with Iran’s seventeen largest cities (those with population > 0.5 million). Aside from the far northwest, and far southeast, each city is adjacent to either localized or more regional areas of vegetation decline during this interval. Map prepared in ArcGIS 10.4.1 (https://www.esri.com/en-us/about/about-esri/overview).

When the NDVI data are mapped (Fig. 7) alongside the cities in Iran with populations in excess of 0.5 million, a possible driver of this spatial variance emerges. The majority of the seventeen largest cities, with the exception of the far northwest and southeast, are located within, or adjacent to, either localized or more regional areas of declining vegetative health. We suggest that this is likely the result of intensified agriculture adjacent to these growing cities, and that groundwater changes may be a key driver. There has been a significant drop in the groundwater table across Iran, which, together with increasing energy consumption, has driven the relationship between agricultural water and energy prices^100^. The exploitation of groundwater affects both the availability and quality of water for agriculture and other uses. The rate of groundwater depletion across Iran has recently been quantified^104^. They reported that the impact of depletion in Iran’s groundwater reserves is negatively affecting ~ 77% of Iran’s land area, together with growing soil salinity, and increasing frequency and extent of land subsidence. Meteorological-hydrological droughts have intensified the rate of depletion of groundwater reserves. In particular, the rate of groundwater overdraft in central Iran is categorized as high to very high and extreme. Even if water can be extracted still, it may be of poor quality; for instance^105^, reported degraded groundwater quality for aquifers in the deserts of central Iran. Due to high concentrations of some anions (e.g., SO_2_^−4^ and Cl^−^) and cations (e.g., K^+^ and Na^+^), the water has been categorized as not suitable for drinking for humans. Extremely high values of electrical conductivity mean that the suitability of regional ground water for agriculture use is also compromised. Illegal groundwater pumping, mainly for regional agricultural use, during recent years has degraded groundwater quality due to saline water intrusion from eastern areas (central Kavir desert and salt lakes) and connate water input from deeper aquifers. Renewed focus on the utilization of groundwater especially in the face of spatially-variable changes in the present precipitation regime seems a vital priority.

Nationwide evaluations of this kind require accurate large-scale datasets that are most effectively extracted via remote sensing. We strongly advocate the use of satellite-derived data for delivering robust information to government policymakers and the scientific community to frame future environmental goals^106,107^. We have shown that large-scale assessments of the extent of land at risk of potential degradation and desertification can be efficiently analyzed via remote-sensing and such activities yield important primary data for policymakers. Crucially, with data such as these, we have demonstrated that the issue of scale, both spatially and temporally, is important in exploring large-scale data such as these. Even if annual temporal changes may not be significant, they may mask important seasonal changes in both drivers and outcomes of desertification, and nationwide syntheses may prove an over-simplification of considerable local and regional variance in landscape stimuli and response. Strategic plans aimed at achieving sustainability must, therefore, consider potential effects of local patterns of imminent climate change and the variations in the responses of biogeochemical cycles.

## Conclusion

Remote sensing data and GIS are essential tools for the evaluation of large-scale desertification, for identifying key factors driving degradation of soils and vegetation, and for the generation of desertification risk maps^108–110^. A key advantage of such data is the capacity to utilize and combine various remote sensing data at different resolutions and likewise, for conducting analyses at different spatial scales.

In this research, a range of remote sensing and climate indices were applied to assess the potential for land degradation and desertification across Iran for the period 2001–2015. Based on the results, the indices we used highlight that Iran is characterized by sparse and poor vegetation cover, which predisposes it to degradation and desertification. The combination of these indices suggests that 68% of Iran is characterized by high and very high desertification potential.

At a nationwide, annual scale, trends in both climatic parameters and vegetation indices have not changed significantly over the 15-year study period, but disaggregating the data seasonally, and on a pixel-by-pixel basis, reveals substantial and significant local impacts. Spatial mapping clearly demonstrates substantial variation in vegetation health trends which in some areas is consistent with similar spatial trends in climatic forcings over the same time period, but in some locations is dissociated from natural drivers. Preliminary work here suggests that the regions surrounding Iran’s largest cities, especially in the center of the country may be especially affected. Using remotely sensed data of even higher resolution may enable regional studies to further explore this variance (e.g. Landsat or Sentinel 2 derived vegetation indices, Global Precipitation Measurement mission data for precipitation).

Although this work suggests that Iran is at very high risk of desertification, and regionally is likely already experiencing the effects of this, there is a limit to the extent that remote sensing can explore the causes of land degradation beyond simple correlation. We have not, for instance, attempted to dissociate natural vegetation, which may well be highly adapted to thriving in arid conditions, and agricultural crops, some of which may be more prone to water stress. Other observations within these data merit further study, such as the decoupling of climate and vegetation health during autumn, and the inversion of the precipitation/vegetation forcing during winter. Future work exploring the processes and causal relationships of changing vegetative health in dryland regions such as Iran will prove crucial in the future under the impacts of a changing climate.
